# Diagnosis and management of elevated intracranial pressure in the emergency department

**DOI:** 10.1186/s12245-023-00540-x

**Published:** 2023-10-13

**Authors:** Sima Patel, Jose Maria-Rios, Amay Parikh, Okorie Nduka Okorie

**Affiliations:** 1 Department of Critical Care Medicine, AdventHealth Orlando, 601 E Rollins St, Orlando, FL 32803 USA; 2 Division of Neurocritical Care, Department of Critical Care Medicine, AdventHealth Orlando, 601 E Rollins St, Orlando, FL 32803 USA

**Keywords:** Intracranial pressure, Emergency department, Intracranial hypertension, Elevated intracranial pressure, Traumatic brain injury

## Abstract

**Background:**

Elevated intracranial pressure is a devastating complication of catastrophic brain injury. Intracranial hypertension is commonly seen in neurologic injury secondary to traumatic brain injuries. Uncontrolled pressures can lead to permanent neurologic damage, but acute medical management is often overlooked when pursuing surgical management options that may not always be indicated.

**Discussion:**

Traumatic brain injury is the leading cause of death in patients with severe neurologic injury. Diagnosing elevated intracranial pressures is imperative in initiating prompt treatment to reduce secondary central nervous system injury, morbidity, and mortality. Although the initial injury to the brain is typically irreversible, intracranial pressure control can assist in salvaging the remaining brain tissue from additional damage. We will discuss the initial medical and surgical management of traumatic brain injury to prevent further neurologic deterioration and reduce mortality.

**Conclusion:**

Recent literature has reported several methods to detect elevated intracranial pressure easily and studies describing multiple treatment modalities. These investigations suggest that early detection and timely treatment of intracranial hypertension are beneficial in reducing mortality.

## Background

Elevated intracranial pressure (ICP) is a topic that inspires self-doubt and fear among physicians in the acute setting. Elevated ICPs can result from many primary pathologies, such as hydrocephalus, intracranial infections, intracerebral hemorrhages, intraventricular hemorrhages, traumatic brain injury (TBI), brain tumors, and many more [1]. In addition, uncontrolled or untreated ICPs can lead to cerebral hypoperfusion, herniation, worsening edema, or death [1–3]. This review aims to identify elevated intracranial pressures and present evidence-based treatment methods to begin the process of reducing ICP to avoid secondary neurologic insult in the emergency department (ED).

## Methods

### Data sources and search

We performed a PubMed, Ovid, Clinical Key, Cochrane Library, Web of Science, and UpToDate search for published elevated intracranial pressure articles, using the following search terms: intracranial pressure physiology, emergency department and elevated intracranial pressure, elevated intracranial pressure and neurosurgery, elevated intracranial pressure and neurocritical care, intracranial pressure and Ketamine, intracranial pressure and optic nerve sheath.

### Study selection

Prospective, observational, and cross-sectional data studies, systemic reviews, meta-analyses, and emergency medicine evaluation guidelines were included. We analyzed 102 papers from 2016 to 2022 and excluded 51 papers. Exclusion papers focused on long-term management for elevated intracranial pressures that were outside the scope of emergency medicine and acute management of ICP. Only cases with elevated intracranial pressures in the acute setting were cited. Eligible articles were published in English.

## Discussion

### Physiology

**Fig. 1:**
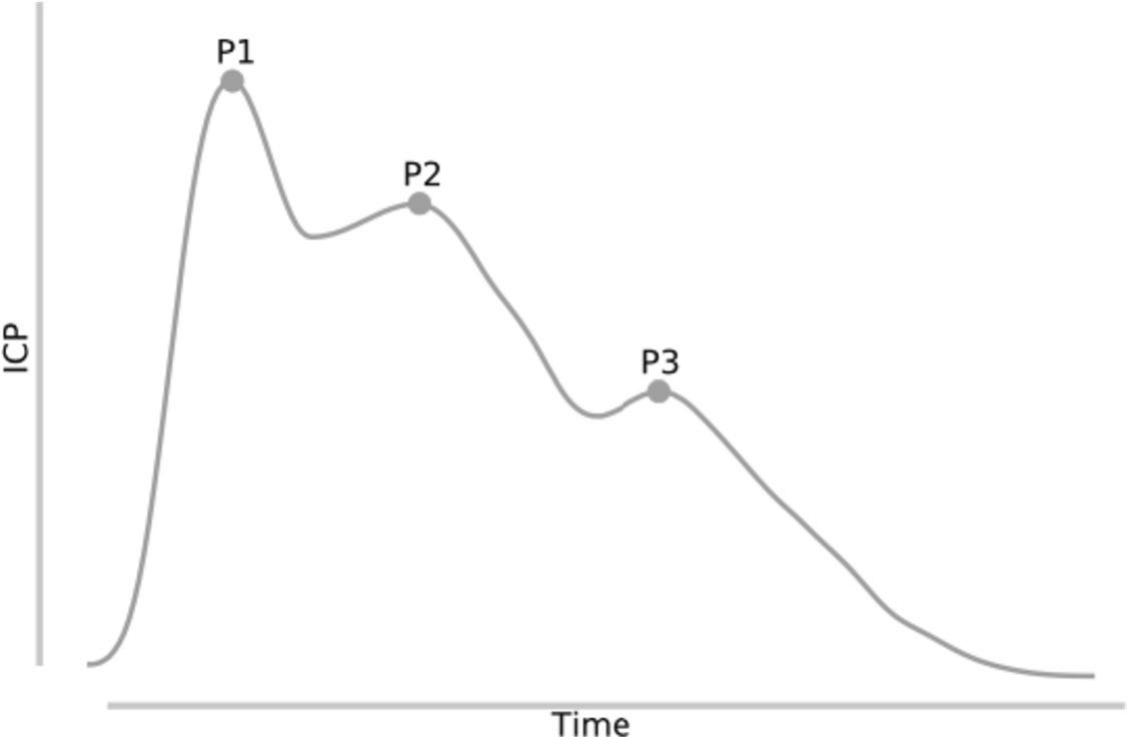
ICP pulse waveform represented by three peaks (P1, P2, P3)

Intracranial hypertension is defined as a sustained (> 5 min) elevation of ICP above 22 mmHg [4]. Typically, normal ICPs are ≤ 15 mmHg in adults. Although ICP is the pressure within the skull, it directly correlates with the cerebrospinal fluid pressure. Notably, ICP measurements demonstrate a pulsatile signal, influenced by the cardiac cycle. The signal is represented by three peaks in the waveform noted in Fig. 1 [1].

The intracranial space is a closed system composed of three components: the parenchymal tissue, cerebrospinal fluids (CSF), and the blood supply [5]. Given brain volume is mostly a fixed factor, cerebral blood flow and production/absorption of cerebrospinal fluids (CSF) are the main contributors to ICP fluctuations [1]. When ICP rises, the distal cerebral arterioles vasodilate to lower cerebral vascular resistance (CVR) to prevent ICP and cerebral blood flow (CBF) elevation [1]. The arterial blood pressure increases if this initial compensation mechanism cannot sustain adequate CBF [1]. Both of these mechanisms will increase cerebral blood volume (CBV); consequently, the ICP will rise until CBF is terminated. This manifestation is how secondary CNS insult occurs following a primary injury. Herniation results from increased intracranial compartment pressures leading to parenchymal tissue shifts that compress or displace cranial nerves or the brainstem [4]. Vascular compression can lead to ischemia or infarction, leading to edema and further brain tissue compromise [4]. Direct ICP measurement requires invasive monitoring, which will be discussed later.

### Diagnosis

#### History and physical exam

A thorough history and physical exam are foremost in starting the process. Clinical signs of increased ICP include headache, nausea and vomiting, or changes in mental status (i.e., confusion, decreased mental abilities, coma, loss of consciousness) [5]. In the ED, the Glasgow Coma Scale (GCS) or the Full Outline of UnResponsiveness (FOUR) score can help assess patient consciousness quickly (Tables 1 and 2). The FOUR score was developed as an alternative to the GCS when evaluating patients with impaired consciousness and possible neurologic injury, especially in intubated patients who cannot be assessed fully.

**Table 1:** Glasgow coma scale

Behavior	Response	Score
Eye opening	Spontaneously	4
	To speech	3
	To pain	2
	No response	1
Best verbal response	Oriented to time, place, and person	5
	Confused	4
	Inappropriate words	3
	Incomprehensible sounds	2
	No response	1
Best motor response	Obeys commands	6
	Moves to localized pain	5
	Flexion withdrawal from pain	4
	Abnormal flexion (decorticate)	3
	Abnormal extension (decerebrate)	2
	No response	1

**Table 2:** The Full Outline of Unresponsiveness (FOUR) score

Eye response	4 = eyelids open or opened, tracking or blinking to command
	3 = eyelids open but not tracking
	2 = eyelids closed but open to loud voice
	1 = eyelids closed but open to pain
	0 = eyelids remain closed with pain
Motor response	4 = thumbs-up, fist or peace sign
	3 = localizing to pain
	2 = flexion response to pain
	1 = extension response to pain
	0 = no response to pain or generalized myoclonus status
Brainstem reflexes	4 = pupillary and corneal reflexes present
	3 = one pupil wide and fixed
	2 = pupillary or corneal reflexes absent
	1 = pupillary and corneal reflexes absent
	0 = absent pupillary, corneal, and cough reflex
Respiration	4 = not intubated, regular breathing pattern
	3 = not intubated, Cheyne-stokes breathing pattern
	2 = not intubated, irregular breathing pattern
	1 = intubated, breathes above ventilator rate
	0 = intubated breathes at ventilator rate or apnea

Exam findings can also vary when herniation or structural shift occurs. Physical manifestations of uncal herniation can include ipsilateral pupillary dilation, contralateral hemiparesis, or acute loss of consciousness [4]. Also, midbrain herniation can result in ipsilateral hemiparesis [4]. A late manifestation of increased ICP is Cushing’s triad (hypertension, bradycardia, and irregular respiration or apnea) [4]. A baseline physical exam is important to later assess for subtle neurologic changes that may require escalation in therapy.

#### Invasive monitoring

Invasive monitoring with an extraventricular drain (EVD) or an intraparenchymal monitor (IPM) remains the gold standard for ICP monitoring [6]. In patients with TBI, guidelines suggest placing an ICP monitor in those who are comatose (GCS 3–8) after adequate neurologic resuscitation and show either abnormalities in computed tomography of the head (CTH) or meet two of the following three criteria: age > 40 years; systolic blood pressure (SBP) < 90 mmHg; or abnormal posturing [4]. Cerebral perfusion pressure (CPP) can be used as a surrogate for CBF; CPP = MAP–ICP. Data supports maintaining a CPP of 60–70 mmHg to prevent cerebral ischemia. Notably, the complications associated with invasive monitoring include infection, hemorrhage, malfunction, obstruction, or malposition [7, 8].

There is little evidence on the insertion timing of invasive monitoring. Hoffman and colleagues performed a retrospective cohort study and concluded a marginal mortality reduction in early (within 6 to 12 h) invasive monitoring placement versus late [9]. They concluded that early detection of elevated ICP, assisted by invasive monitoring, could result in prompt treatment; however, prospective data is required to assess this outcome further. Given that the ED encounters arriving patients after acute insults occur, functional outcomes could be improved by identifying the need for invasive early monitoring, considering the risks and benefits, or facilitating early neuro-critical care admission.

#### Quantitative pupillometry

Noninvasive monitoring techniques have been introduced in the detection of elevated ICP. Examining the pupillary light reflex (PLR) has always been an integral part of a comprehensive neurologic exam. Traditionally performed with a penlight, PLR has been replaced with an automated pupillometer assessment [10]. Examination of pupillary reflexes utilizing a penlight developed descriptive terms such as sluggish, brisk, nonreactive, or fixed [10]. Not to mention, it was difficult to assess the circumstances in which the exam took place. In an effort to minimize subjective language and develop a unified, standard definition, automated pupillometer was introduced.

This tool provides an objective measure of pupillary constriction, dilation velocities, pupil size, and latency [10]. The tool uses an algorithm to calculate an index called “NPi” or neurologic pupil index, a measure of the pupil’s reactivity. The numerical range is from 0 to 5, where a value greater than or equal to 3 indicates normal activity [10]. An abnormal NPi (less than 3) can be associated with damage to the third cranial nerve or possibly damage to the brain [10].

Several studies have linked the association between poor NPi and elevated ICP. McNett et al. suggest that an increased ICP may be revealed as a decreased NPi and reduced contraction velocities, with minimal change in the pupillary size [11]. Many other studies detected an inverse relationship between ICP and NPi values [10]. Sameer et al concluded that patients with a lower PLR, lower NPi, reduced constriction velocity, and slower dilation velocities had increased ICP values (> 15 mmHg) [10]. The use of a pupillometer to obtain quantitative data of PLR using NPi is useful in the early detection of elevated ICP.

#### Point of care ultrasound (POCUS)

Point of care ultrasound (POCUS) has been studied for use in detecting elevated ICP via measurement of the optic nerve sheath diameter (ONSD). As ultrasound is readily available in most emergency departments, assessing the ONSD can quickly offer additional information for appropriate management. The ONSD, an indirect measurement of raised ICP, can be measured via brain CT or an ocular ultrasound [12]. The subarachnoid space of the optic nerve sheaths and the brain are anatomically connected, thus allowing CSF to circulate from the perichiasmatic cistern to the ocular areas [13]. As the ICP increases, the pressure drives a large quantity of CSF toward the orbital spaces, resulting in an enlargement of the ONSD [13]. Literature also compares ONSD to eyeball transverse diameter (ETD) by ultrasound to predict elevated ICP. Du and colleagues found the correlation between ultrasounds-ONSD/ETD ratio, ultrasound-ONSD, CT-ONSD/ETD ratio, and ICP were 0.613, 0.498, and 0.688 (*P* < 0.05) [14]. They concluded that the ratio of ONSD to ETD by ultrasound may be reliable for predicting elevated ICP in patients with TBI [14]. A prospective blinded observational study in 2011 found that bedside ONSD measurements, when performed by a neurointensivist, accurately identified ICP greater than 20 mmHg when ONSD was found equally or greater than 0.48 cm [15]. The measurement of ONSD may be useful for assessing ICP when instability precludes travel to CT or when invasive monitoring methods are unavailable.

#### Computed tomography of the head (CTH)

Fernando and colleagues evaluated multiple CT signs for elevated ICP, including basal cistern compression, midline shift of varying degrees (> 5 mm or > 10 mm), and the Marshall classification system (Table 3) [3].

**Table 3:** The Marshall classification system of TBI

Class		Description
I	Diffuse injury I (no visible pathology)	No visible pathology was seen on CTH
II	Diffuse injury II	-Midline shift of 0 to 5 mm -Basal cisterns remain visible -No high or mixed-density lesions > 25 cm^3^
III	Diffuse injury III (swelling)	-Midline shift of 0 to 5 mm -Basal cisterns compressed or completely effaced -No high or mixed-density lesions > 25 cm^3^
IV	Diffuse injury IV (shift)	Midline shift > 5 mm No high or mixed-density lesions > 25 cm^3^
V	Evacuated mass lesion	Any lesion evacuated surgically
VI	Non-evacuated mass lesion	-High or mixed-density lesions > 25 cm^3^ -Not surgically evacuated

They calculated the sensitivities and specificities of each quality for diagnosing elevated ICP shown in Table 4 [3]. The diagnostic sensitivity decreased from a Marshall Class of three (80.6%, 63.5% to 90.9%) to five (45.1%, 28.5% to 76.4%) [3]. However, the diagnostic specificity increased from a Marshall Class of three (59.9%, 40.9% to 76.4%) to a class of five (83.5%, 70.4% to 91.5%) [3].

**Table 4:** Sensitivities and specificities of diagnosis of elevated ICP

Quality	Sensitivity	Specificity
Absence or compression of basal cisterns on CTH	85.9% (95% confidence interval 58.0% to 96.4%)	61% (29.1% to 85.6%)
Any midline shift	80.9% (64.3% to 90.9%)	42.7% (24.0% to 63.7%)
Severe midline shift (> 10 mm)	20.7% (13.0% to 31.3%)	89.7% (77.5% to 95.2%)

Newer CT scoring systems have been discussed since the Marshall system, such as the Rotterdam score, Stockholm score, and Helsinki CT score [16]. Recent studies have compared many of these scores in outcome predictors of mortality instead of diagnostic utility [16]. More research is required to accurately depict whether these scoring systems provide any diagnostic information that directly correlates with elevated ICPs.

Other devices that can aid in the diagnosis of elevated ICP include electroencephalography, transcranial Doppler, Brain4Care, and Vittamed 205. However, due to limited availability, ease of use, institutional and technical limitations, they were not discussed in detail.

### Management

**Fig. 2:**
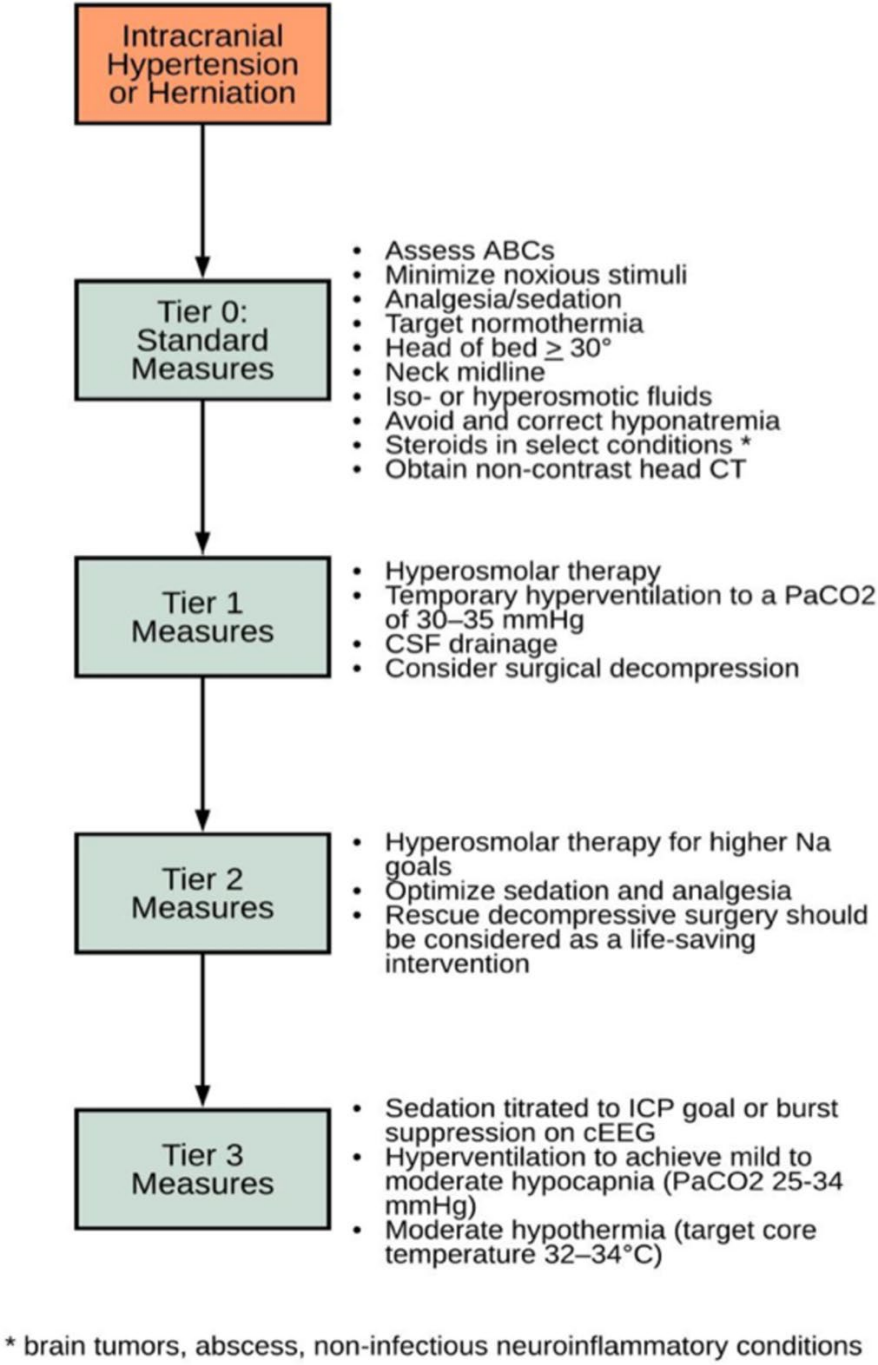
Emergency neurologic life support intracranial hypertension and herniation algorithm

Management of increased intracranial pressure should be tackled systematically in the emergency department. The Emergency Neurological Life Support (ENLS) created a simplified tiered approach for management (Fig. 2).

Initiating sedation and analgesia helps reduce noxious stimulation that may increase ICP [4]. In addition, elevating the head of the bed more than 30° and keeping the neck midline assists cerebral venous drainage [4].

### Hyperosmolar therapy

Hyperosmolar therapy, including mannitol or hypertonic saline (HTS), has equally shown benefits in lowering ICP. Mannitol is given as a 0.5–1 g/kg IV bolus via a peripheral intravenous line over 5–15 min. This process can be repeated every 4–6 h. Monitoring adequate dosing can be based on the osmolar gap; a gap > 20 mOsm/kg has shown no benefit. Hypertonic saline is available from 2 to 23.4% and can be administered as a bolus alone or with mannitol. Concentrations of HTS greater than 7.5% should be given through a central venous catheter. Concentrations less than 7.5% can be bolused via a peripheral line; however, infusions should be given in a large vessel. Generally speaking, equimolar dosing is recommended during any hyperosmolar agent administration. Dosing is variable throughout many studies and institutional practices/protocols differ depending on what is available. Sodium concentration, osmolarity, and bolus dosing can be seen in Table 5. As far as continuous infusions, the rate depends upon the targeted sodium as no specific rate is recommended. However, to reach the desired serum sodium concentration goal, one can implement the Adrogue-Madias formula (Change in serum sodium (Na) = infusate Na (mEq/L)–serum Na (mEq/L)/ total body water + 1) to calculate the rate of whichever strength saline agent they are using [17, 18]. With hypertonic saline, serum sodium concentration should be monitored every 4–6 h, and the concentration should be kept at < 160 mEq/L [4]. Whether continuous infusion of 3% HTS benefits ICP control versus bolus therapy remains controversial [4]. Ultimately, there is no clear recommendation for using HTS over mannitol for patients with TBI [19].

**Table 5:** Sodium concentration, osmolarity, and bolus dosing for hyperosmolar agents [4, 20, 21]

Hyperosmolar agent	Sodium Concentration (mEq/L)	Osmolarity (mOsm/L)	Bolus range dosing
Mannitol 20%	n/a	1098	0.5–1.0 g/kg
Mannitol 25%	n/a	1375	0.5–1.0 g/kg
3%	513	1027	1.4–2.5 mL/kg
7.5%	1282	2566	1 mL/kg
23.4%	4004	8008	30 mL

^*^mEq milliequivalents, *mOsm* milliosmole, *L* liter, *g* gram, *mL* milliliters, *kg* kilogram

#### Antiepileptics

Seizure activity (i.e., gross seizures, subclinical, and post-traumatic epilepsy) can be seen in those with increasing ICP. Seizure activity can increase cerebral metabolic demand and worsen ICP [22]. Anticonvulsants should be considered in those with increased ICP, especially depending on the etiology. Patients with TBI, intracranial hemorrhages, subarachnoid hemorrhage, and large volume ischemic injuries would benefit from prophylactic anticonvulsant therapy. Phenytoin and levetiracetam are the most commonly used agents [22]. Prophylaxis has been shown to prevent early seizure activity but not post-traumatic epilepsy [22]. There is no evidence to suggest loading dose therapy is beneficial in preventing seizures or progression to epilepsy.

#### Induction agents and sedation

Fentanyl has been used in premedication to blunt increases in ICP related to laryngotracheal stimulation in rapid sequence intubation (RSI) [23, 24]. In a normotensive/hypertensive patient, an intravenous bolus of fentanyl (2–3 mcg/kg) 3 min prior to induction is recommended [23, 25]. Fentanyl is hemodynamically neutral; however, it can decrease MAP and CPP when given in bolus doses [23]. Etomidate is commonly used as an induction agent in patients with TBI because it can decrease cerebral blood flow and cerebral metabolic demand while preserving cerebral perfusion pressure.

If ICP remains a concern, increasing sedation can assist in management. Propofol can decrease cerebral metabolic demand (CMRO2) and CBV [26]. A bolus dose of 1–2 mg/kg can be administered, followed by a continuous infusion to a maximum of 200 μg/kg/min in ventilated patients [4]. However, propofol has an increased propensity to cause systemic hypotension. A MAP of 80 to 110 mmHg should be targeted to maintain a CPP greater than 60 mmHg [24]. Vasopressors can be implemented to maintain cerebral perfusion pressure goals. Tier 3 of ENLS focuses on the most aggressive level of management. This tier titrates sedation to ICP goals or burst suppression on continuous electroencephalogram (cEEG). Pentobarbital can be administered and titrated to both goals (bolus 5–15 mg/kg over 30 min to 2 h, followed by a maintenance infusion of 1–4 mg/kg/h).

Midazolam has a neutral hemodynamic profile and can lower systemic blood pressure, thus reducing CPP [23]. Another concern surrounding midazolam is its ability to accumulate in adipose tissue over time, resulting in delayed awakening [23, 24]. Midazolam has also been shown to increase ventilatory days, prolonged coma, and ICU length of stay [23]. However, its anticonvulsant and anxiolytic properties can be beneficial in certain cases [23, 24].

The use of Ketamine has been controversial in patients with head injury [24]. It was thought that its positive effects on sympathetic stimulation might cause a rise in ICP via stimulation and exacerbate the underlying condition [27]. However, when Ketamine is used alongside a y-aminobutyric acid (GABA) receptor agonist, the ICP rise may not occur [28]. Ketamine may also be considered beneficial by increasing cerebral perfusion through a transient increase in systemic blood pressure [28]. Kumar and colleagues’ literature review determined that Ketamine can be used safely in patients with TBI, although no large clinical trials have been done [28]. Ketamine also positively affects intraocular pressure (IOP) in a dose-dependent fashion. Usually, doses less than 4 mg/kg decreased IOP [28]. However, due to limitations and weaknesses, several retrospective and prospective studies have not been able to conclude a solid recommendation on ketamine use in this patient population [29].

#### Paralysis

There is not enough current evidence to recommend rocuronium over succinylcholine for rapid sequence intubation (RSI) in patients with TBI [23]. Notably, it was shown that sustained paralysis with rocuronium could prevent repeat neurologic exams [25]. Patients who receive rocuronium are also administered less sedation and analgesia post-intubation because paralysis makes it appear the patient is sedated [23]. In addition, the rapid offset of succinylcholine allows for early neurologic exams [23].

Table 6 summarizes the physiologic effects of induction or sedating medications with key points for selecting agents for patients.

**Table 6:** Medication physiologic effects and key points [23, 24]

Medication	Intracranial pressure (ICP)	Cerebral blood flow (CBF)	Cerebral blood volume (CBV)	Cerebral metabolic rate (CMR)	Keypoints
Barbiturates	↓	↓	↓	↓	
Benzodiazepines	↓ or NC	↓	↓	↓	-Neutral hemodynamic profile -anxiolytic and anticonvulsant effects
Etomidate	↓	↓	↓	↓	-Mild hemodynamic profile -For the normotensive/hypertensive patient
Ketamine	↑	↑	↑	↑ or NC	-Can be neuroprotective without increasing cerebral oxygen consumption -Has sympathetic stimulation properties that may aid in increasing MAP and CPP -For the hypotensive patient
Opiates	NC	NC	↓ or NC	NC	-Neutral hemodynamic profile -Can blunt the sympathetic response of elevated MAP and heart rate in RSI -For the normotensive/hypertensive patient
Propofol	↓	↓	↓	↓	-Decreases cerebral metabolic demand and cerebral blood volume -For the normotensive/hypertensive patient

^*^Decrease (↓), increase (↑), no change (NC)

#### Ventilation

While additional therapies are being implemented, a brief moment of hyperventilation (< 2 h) to a PaCO2 of 30–35 mmHg may be considered [24]. In addition, hyperventilation for a goal of mild to moderate hypocapnia (PaCO2 25–34 mmHg) may be considered in patients who have failed other management strategies [4]. However, hyperventilation for more than 6 h is unlikely beneficial and may exacerbate ischemic injury due to cerebral vasoconstriction [4, 24]. Ideally, cerebral oxygen monitoring, such as jugular venous oximetry or brain tissue oxygen monitoring, should be implemented to monitor for cerebral ischemia.

#### Surgical intervention

Notably, early surgical decompression is recommended by ENLS if tier 1 is ineffective in reducing ICP [4]. However, if surgery is inappropriate, tier 2 should be the next step in management [4]. Rescue decompressive surgery is the last step in tier 2 as a lifesaving intervention because tier 3 therapy implies the patient is no longer a candidate for surgery [4].

A decompressive craniotomy (DC) aims to remove a portion of the skull, allowing the brain room for expansion to decrease ICP and improve blood flow and cerebral oxygenation [22]. Larger craniotomies (> 12 × 15 cm or 15 cm in diameter) are preferred over smaller sized to decrease mortality and improve neurologic outcomes [22, 30]. Cochrane Library reviewed three trials with 590 participants and concluded that decompressive craniotomy decreases mortality; however, the long-term neurologic outcomes are controversial [31]. Given that most neurologic injuries focus on long-term functional outcomes, a family discussion on care goals and quality of life would be warranted. Future studies should focus on the most appropriate timing for DC, ideal techniques, and patient long-term functional outcomes.

## Conclusion

Diagnosis of elevated intracranial pressure and prompt initiation of management begins in the emergency department to optimize patients prior to intensive care admission or operating room time. Utilization of noninvasive diagnostic measures such as a POCUS or CT head can clarify the need for escalation to invasive diagnostic measures such as an EVD or IPM. Starting tiered therapy from hypertonic therapy to surgical consultation may improve end functional outcomes. Timely interventions and the early involvement of a multidisciplinary team can help ensure patients receive adequate care and reduce mortality.

## Data Availability

Not applicable.

## Abbreviations

ICP: Intracranial pressure
TBI: Traumatic brain injury
ED: Emergency department
CVR: Cerebral vascular resistance
CBF: Cerebral blood flow
US: United States
GCS: Glasgow Coma Scale
FOUR: Full outline of UnResponsiveness
EVD: Extra ventricular drain
IPM: Intraparenchymal monitor
CTH: Computed tomography of the head
SBP: Systolic blood pressure
CPP: Cerebral perfusion pressure
MAP: Mean arterial pressure
POCUS: Point of care ultrasound
ONSD: Optic nerve sheath diameter
CSF: Cerebrospinal fluid
ETD: Eyeball transverse diameter
ENLS: Emergency neurologic life support
HTS: Hypertonic saline
RSI: Rapid sequence intubation
CMRO2: Cerebral metabolic demand
cEEG: Continuous electroencephalogram
GABA: y-aminobutyric acid
IOP: Intraocular pressure
RSI: Rapid sequence intubation
DC: Decompressive craniotomy
mEq: Milliequivalents
mOsm: Milliosmole
L: Liter
g: Gram
mL: Milliliters
kg: Kilogram
Na: Sodium
PLR: Pupillary light reflex
NPi: Neurologic pupil index
